# Nickel Electrocatalysts Obtained by Pulsed Current Electrodeposition from Watts and Citrate Baths for Enhanced Hydrogen Evolution Reaction in Alkaline Media

**DOI:** 10.3390/ma18122775

**Published:** 2025-06-12

**Authors:** Raluca Bojîncă, Roxana Muntean, Rebeca Crişan, Andrea Kellenberger

**Affiliations:** 1 Faculty of Chemical Engineering, Biotechnologies and Environmental Protection, Politehnica University Timişoara, Piata Victoriei No. 2, 300006 Timişoara, Romania; raluca.bojinca@student.upt.ro (R.B.); rebeca9803@gmail.com (R.C.); 2 Department of Materials and Manufacturing Engineering, Politehnica University Timișoara, Piata Victoriei No. 2, 300006 Timişoara, Romania; roxana.muntean@upt.ro

**Keywords:** hydrogen evolution reaction, alkaline media, nickel electrodeposition, citrate bath, pulsed current electrodeposition

## Abstract

Efficient and low-cost electrocatalysts for the hydrogen evolution reaction (HER) in alkaline media are essential for sustainable hydrogen production. In this study, Ni electrocatalysts were deposited on pencil graphite using a simple one-step pulsed current electrodeposition method, from both acidic Watts and alkaline citrate baths. The influence of bath type and electrodeposition parameters—current density and temperature—on catalyst morphology and performance for HER was systematically investigated by scanning electron microscopy and electrochemical methods. Linear sweep voltammetry, chronopotentiometry, and electrochemical impedance spectroscopy (EIS) were used to evaluate the electrocatalytic activity, stability, and HER mechanism. The best catalytic performance was achieved for the Ni electrocatalyst deposited from the citrate bath at 50 mA cm^−2^ and 40 °C, showing an exchange current density of 0.93 mA cm^−2^, a Tafel slope of −208 mV dec^−1^, and overpotentials of −210 mV and −386 mV at 10 and 100 mA cm^−2^, respectively, in 1 M KOH solution. Chronopotentiometry confirmed improved stability and an overpotential reduction of approximately 92 mV as compared to pure Ni, while EIS revealed the lowest charge transfer resistance. It was shown that the electrocatalysts deposited from the citrate bath outperform those from the Watts bath, and electrodeposition at 40 °C is optimal for achieving the highest electrocatalytic activity for HER.

## 1. Introduction

In recent years, the focus on renewable energy technologies has significantly propelled research into advanced electrolysis systems, particularly on anion exchange membrane (AEM) electrolyzers, which possess substantial potential for efficient hydrogen production. The core reactions at the cathode and anode are the hydrogen evolution reaction (HER) and oxygen evolution reaction (OER), respectively, both being successfully catalyzed by the benchmark electrocatalysts based on platinum, iridium, or ruthenium [1,2]. Besides these, nickel and its alloys have emerged as promising non-precious metal electrocatalysts for HER due to their remarkable intrinsic properties and electrochemical stability under operational conditions, especially in alkaline media [3,4]. Its abundance, cost-effectiveness, and favorable catalytic activity make it an attractive alternative to noble metal catalysts [5,6], which, despite their high efficiency, are hindered by scarcity and expense. In alkaline environments, the HER process is often limited by the sluggish kinetics of water dissociation. In this regard, to further enhance the activity and performance, researchers have explored various strategies, including alloying nickel with other metals [7,8] to form compounds like sulfides [9,10], selenides [11], phosphides [12] and oxides [13,14] as well as engineering nanostructures to increase the electrochemically active surface area [15]. Feng et al. reported a nanoscale electrocatalyst composed of NiS_2_ and Ni_3_C@C, presenting exceptional stability and HER performance superior to many traditional catalysts [9]. Similarly, Liu et al. suggested nickel phosphides, which facilitate hydrogen adsorption and desorption, thereby accelerating reaction rates during HER, highlighting them as effective catalysts for alkaline electrolysis [12]. Sadhanala et al. illustrated that the Ru-doped nickel foam exhibits enhanced performance due to synergistic effects, improving stability and catalytic efficiency in alkaline environments [16]. In addition to these materials, nickel-based nitrides have attracted considerable interest due to their promising electrochemical properties. The study of Wang et al. demonstrated that nitride materials, particularly nickel–cobalt nitrides, show excellent catalytic activity for both HER and OER, underscoring their versatility in alkaline electrolysis setups [17]. Their effective performance is attributed to improved electronic properties and enhanced water dissociation kinetics, which are vital for efficient hydrogen production. One of the notable approaches to improving nickel-based catalysts lies in the development of bimetallic and heterostructured materials. For instance, the combination of nickel with transition metals such as molybdenum has shown significant enhancement in HER activity [18,19]. The study of Fernández-Valverde et al. demonstrated that Ni-Mo electrocatalysts possess advantageous electrochemical behavior in alkaline solutions, establishing them as references in commercial alkaline electrolyzers due to their superior efficiency compared to pure nickel [20]. Furthermore, incorporating heteroatoms into nickel-based structures has proven to be an effective strategy to increase overall HER performance, as discussed by Deng et al., which optimizes the electronic structure and stability of the catalysts [21]. These findings are supported by Wang et al.’s study, which showed that synergetic effects between bimetallic phosphide catalysts can significantly reduce overpotentials, thereby enhancing hydrogen production efficiency [22]. Moreover, advancements in the architecture of nickel catalysts have further improved their electrochemical performance. Li et al. reported on the efficient water splitting facilitated by three-dimensional nickel molybdenum phosphide hetero-nanorods, emphasizing that the rough surface texture provided a high density of active sites, enabling rapid electron and proton transfer [23]. Such three-dimensional structures allow for effective mass transport and increased active surface area, leading to enhanced catalytic efficiency. Additional studies indicate that variation of compositions and structures, such as nickel hydroxides or oxides, can facilitate superior catalytic properties, with amorphous metallic nickel being highlighted for its impressive HER activity with minimal overpotential [24].

Among different manufacturing techniques, electrodeposition has been employed to fabricate nickel coatings [25] or particles [26], with controlled textures and porosities, which are crucial for optimizing HER performance. Electrodeposition involves the reduction of Ni^2^^+^ ions from an electrolyte solution onto a conductive substrate under an applied electric potential. The process parameters—such as electrolyte composition, pH, temperature, deposition potential, and current density—play pivotal roles in determining the nucleation and growth mechanisms of Ni, thereby affecting the microstructure and catalytic properties of the deposited layer [27,28,29]. A promising technique for tailoring the microstructure of electrodeposited materials is pulsed current (PC) electrodeposition. By alternating the applied current between a high value during the on time (t_on_) and zero or low current during the off time (t_off_), this method enhances nucleation rate and offers better control over growth, leading to finer-grained deposits with improved structural uniformity and electrochemical performance compared to conventional direct current electrodeposition [30]. Optimizing pulse parameters allows obtaining nanocrystalline materials with superior catalytic activity due to their favorable morphology, increased surface area, improved charge transfer kinetics, and more active sites available for the HER [31,32].

A significant role in enhancing the activity, stability, and electrochemical properties of the catalysts is played by the support material. Carbon-based supports such as graphene, carbon nanotubes, or carbon nanofibers are commonly used due to their high electrical conductivity, large surface area, and chemical stability across various pH ranges. Among them, pencil graphite electrodes (PGE) are frequently used in sensor applications and electrocatalysis [33,34], including HER [32,35], based on their low cost, accessibility, and suitability as a scalable platform for preliminary electrochemical studies.

In this study, we report on nickel deposits obtained on PGE substrate by pulsed current electrodeposition, from acidic Watts and alkaline citrate baths, with enhanced electrocatalytic activity for HER in alkaline media. We investigated the effect of temperature and different bath compositions on the electrocatalytic activity for HER. Field emission scanning electron microscopy (FE-SEM) and energy dispersive X-ray spectroscopy (EDAX) were employed to investigate surface morphology and chemical composition. The electrocatalytic performance for HER was evaluated and compared using linear sweep voltammetry (LSV) and kinetic parameters extracted from the Tafel plots. Additionally, the overpotential required to achieve current densities of 10, 50, and 100 mA cm^−2^ served as an indicator for comparing the activity of all Ni-PGE electrocatalysts. Stability tests were performed at constant current densities of 10 and 100 mA cm^−2^. In addition, electrochemical impedance spectroscopy (EIS) was used to gain deeper insight into the electrode behavior and underlying HER mechanism at various overpotential values. The results demonstrate that Ni electrocatalysts prepared from an alkaline citrate bath at an optimal deposition temperature of 40 °C exhibit superior performance for HER. These findings provide a foundation for the development and design of HER electrocatalysts with improved electrocatalytic activity in alkaline media, utilizing a simple, one-step electrodeposition approach and an affordable and available substrate.

## 2. Materials and Methods

### 2.1. Preparation of Ni-PGE by Pulsed Current Electrodeposition

Nickel electrodeposition on PGE was carried out via pulsed current electrodeposition, from citrate and Watts bath, respectively, with the compositions described in Table 1. For the electrodeposition, a three-electrode cell was used, with PGE as working electrode, a silver—silver chloride (Ag/AgCl in 3 M KCl, BASi^®^, West Lafayette, IN, USA)—as reference electrode, and a Pt wire as counter electrode. To obtain a constant surface area for Ni electrodeposition, the PGEs were isolated with Teflon band, leaving a controlled length of 20 mm in contact with the electrolyte solution. Before use, the PGEs were cleaned in ethanol using an ultrasound bath and finally rinsed with distilled water.

**Figure 1:**
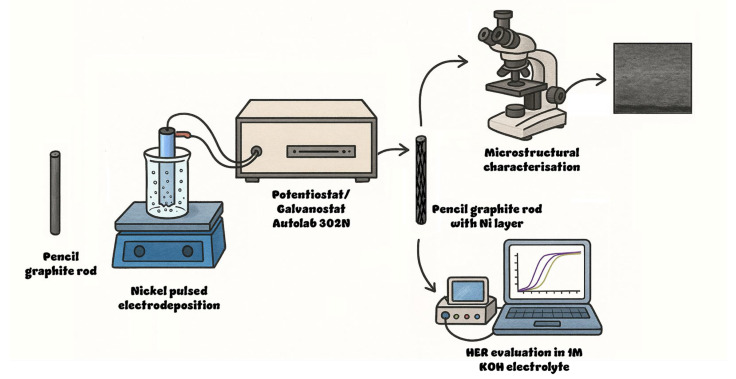
Schematic representation of the electrode fabrication and testing.

Constant cathodic current pulses of 25 and 50 mA cm^−2^ were applied using an Autolab 302N potentiostat/galvanostat (Metrohm Autolab, Utrecht, The Netherlands) with the following deposition parameters: on time *t*_on_ = 0.1 s, off time *t*_off_ = 0.5 s, and 500 cycles, resulting in a duty cycle of 16.67%. Several electrodes were prepared by electrodeposition at three different temperatures, namely 20, 40, and 60 °C, while keeping the pulse parameters as stated. The fabrication process is described in Figure 1.

The citrate bath was prepared using nickel sulfate hexahydrate (NiSO_4_·6 H_2_O, Chimreactiv, p.a.) and trisodium citrate pentahydrate (Na_3_C_6_H_5_O_7_·5H_2_O, Chimreactiv, p.a.) with the composition given in Table 1. After preparation, the pH was adjusted to 8 by dropwise addition of ammonia.

In the citrate bath, trisodium citrate acts as a complexing agent, forming nickel–citrate complexes [36] that influence the nucleation and growth of nickel deposits. This results in smoother, more uniform Ni layers with finer crystal structure than those obtained from the Watts bath [37]. Nickel electrodeposited from citrate baths typically exhibits a compact, globular surface and nanocrystalline microstructure [28,38]. In the Watts bath, nickel sulfate hexahydrate (NiSO_4_·6 H_2_O, Chimreactiv, p.a.) and nickel chloride hexahydrate (NiCl_2_·6 H_2_O, Merck, EMSURE^®^, ACS) provide the necessary nickel ions for the cathodic deposition, while boric acid (H_3_BO_3_ Merck, EMSURE^®^, ACS, ISO, Reag. Ph Eur) acts as a buffering agent to ensure the acidic environment during the electrodeposition process.

### 2.2. Physico-Chemical Characterization of Ni-PGE

The structure and morphology of Ni-PGE were studied using field emission scanning electron microscopy (FE-SEM) on a QUANTA FEG 250 microscope (Quanta FEG 250, FEI, Hillsboro, OR, USA), with the Everhart–Thornley detector (ETD) for secondary electrons (SE). Images were taken in a high vacuum mode with an accelerating voltage of 10 kV and a working distance of 10 mm. The elemental composition was determined by energy dispersive X-ray analysis (EDAX with Apollo SSD detector, EDAX Inc., Mahwah, NJ, USA).

### 2.3. Performance of Ni-PGE for Hydrogen Evolution Reaction

The performance of Ni-PGE for HER was evaluated using a combination of electrochemical methods, including linear sweep voltammetry (LSV), chronopotentiometry (CP), and electrochemical impedance spectroscopy (EIS). All electrochemical tests were carried out on an Autolab 302 N potentiostat/galvanostat, in a three-electrode configuration cell, with Ni-PGE as working electrode, a silver—silver chloride (Ag/AgCl in 3 M KCl)—as reference electrode and a Pt wire as counter electrode. The measured electrode potentials versus the reference were converted to the reversible hydrogen electrode (RHE) using the formula *E*_RHE_ = *E*_Ag/AgCl_ + 0.059 pH + *E*^o^_Ag/AgCl_, where *E*_Ag/AgCl_ is the measured potential, and *E*^o^_Ag/AgCl_ is the potential of the Ag/AgCl electrode (0.210 V for a 3 M KCl solution). The test solution was 1 M KOH (Merck, EMSURE^®^, p.a.).

LSV measurements were recorded in 1 M KOH at a scan rate of 10 mV s^−1^ to obtain the Tafel plots and calculate the kinetic parameters: *b* is the Tafel slope, and *i*_0_ is the exchange current density. CP measurements were conducted in 1 M KOH at a current density of 10 and 100 mA cm^−2^ for 2 h to investigate HER overpotentials and Ni-PGE stability. EIS measurements were carried out over a frequency range of 100 kHz to 10 mHz with an AC voltage amplitude of 10 mV rms. For each spectrum, 60 points were collected using a logarithmic distribution of 10 points per frequency decade. Impedance spectra for each Ni-PGE were measured at five different HER overpotentials ranging from −50 mV to −250 mV. The experimental EIS data were modeled to an equivalent electrical circuit (EEC) using a complex non-linear least-squares Levenberg–Marquardt procedure with the ZView 3.0 software. All electrochemical measurements were repeated on a nickel wire (ϕ = 1 mm, 99.2% Ni), and the HER performance of Ni-PGEs was compared to that of a reference Ni wire electrode.

## 3. Results and Discussion

### 3.1. Pulsed Current Electrodeposition of Ni-PGE

To investigate the effect of bath type and electrodeposition parameters—pulsed current density and temperature—on the electrocatalytic activity for HER, several electrodes were prepared by varying the parameters according to the data presented in Table 2.

In all cases, the pulse parameters were maintained similarly, as described in the Materials and Methods Section. For the Watts bath, a pulsed current density of 25 mA cm^−2^ was not adequate to achieve deposition; therefore, only deposits obtained at 50 mA cm^−2^ were further investigated.

### 3.2. Characterization of Ni-PGE

**Figure 2:**
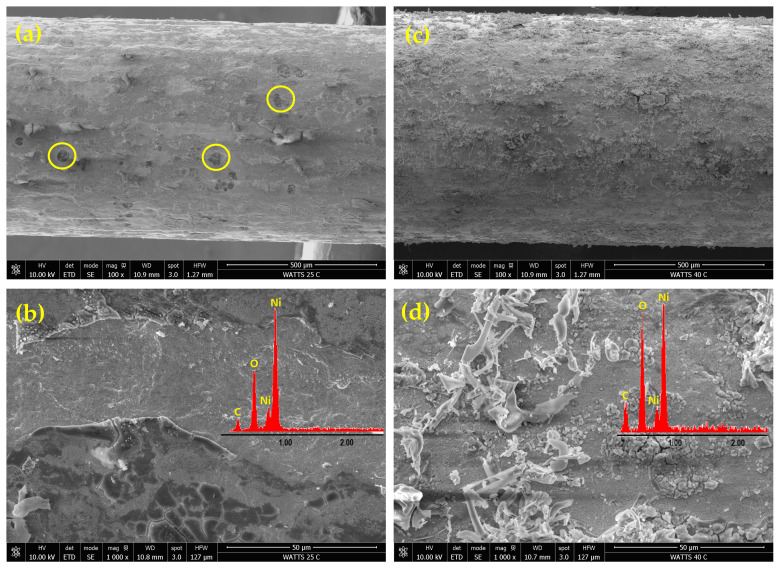
FE-SEM micrographs of nickel deposits on PGE obtained by pulsed current electrodeposition from the Watts bath at 50 mA cm^−2^ current density and the two different temperatures of 25 °C and 40 °C: Ni-PGE-W1 (**a**,**b**) and Ni-PGE-W2 (**c**,**d**). Insets show the results of EDAX elemental analysis.

Figure 2 presents FE-SEM images of Ni deposits obtained from an acidic Watts bath at a current density of 50 mA cm^−2^ and two different temperatures: 25 and 40 °C. The EDAX elemental analysis results are shown as insets in Figure 2b,d. The observed nickel deposit morphology is consistent with nickel electrodeposition from the Watts bath occurring simultaneously with the cathodic HER.

The lower-magnitude FE-SEM images from Figure 2a,c reveal a heterogeneous, relatively compact structure, indicating a low specific surface area. The deposit obtained at 25 °C (Figure 2a) shows a rough texture with visible irregularities and a large number of circular defects (marked as yellow circles) resulting from hydrogen bubbles entrapment or shielded regions where nickel deposition was hindered by gas evolution. When the temperature is increased to 40 °C (Figure 2c), the deposition rate increased, resulting in more uniform coverage, but at the same time, dendritic structures and filament-like growths began to form. In the Watts bath, increasing temperature enhanced the diffusion of Ni^2^^+^ ions towards the cathode and favored rapid growth at existing nucleation sites rather than the formation of new sites. This explains the formation of dendritic or filament-like structures, particularly under high current density conditions. At increased magnification, the FE-SEM images in Figure 2b,d show a layered structure, with a compact base layer and globular morphology. At 25 °C (Figure 2b), numerous cracks are visible in the top layer, caused by internal stresses during deposition. At 40 °C (Figure 2d), a finer grain structure is observed covered by filaments with a dendritic structure.

**Figure 3:**
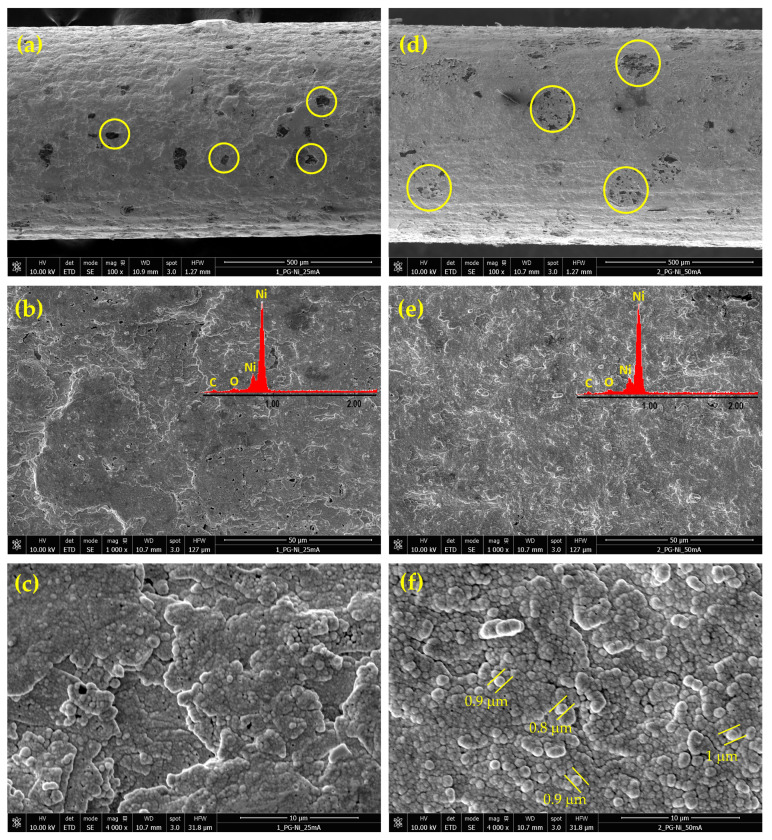
FE-SEM micrographs of nickel deposits on PGE obtained by pulsed current electrodeposition from the citrate bath at 40 °C and the two different current densities of 25 and 50 mA cm^−2^: Ni-PGE-C2 (**a**–**c**) and Ni-PGE-C5 (**d**–**f**). Insets show the results of EDAX elemental analysis.

Figure 3 shows selected FE-SEM images of the Ni deposits obtained from the alkaline citrate bath at 40 °C and two different current densities: 25 and 50 mA cm^−2^.

Similar to the Watts bath, nickel electrodeposition from citrate bath occurs simultaneously with HER. However, in alkaline solution, the deposited nickel acts as an electrocatalyst for HER. Consequently, the FE-SEM images in Figure 3a,d reveal more frequent and larger shielded areas where nickel deposition was hindered due to hydrogen bubbles formation (yellow circles), as compared to the Watts bath. As the deposition current density increased, the current efficiency for nickel deposition decreased due to the intensification of the secondary reaction, and the spots corresponding to hydrogen bubbles became even larger. Simultaneously, higher current density produces a more uniform deposition structure. Comparing Figure 3b,e, it is evident that at low current density, more pores and voids formed, whereas at high current density, the deposit became more uniform and homogeneous. At higher magnification, the nickel deposits in Figure 3f exhibit a homogeneous, globular morphology, with structures approximately 1 µm in size and characteristic of citrate-based alkaline baths [38]. This morphology provides a higher specific surface area compared to deposits from the acidic Watts bath. Unlike the Watts bath, the presence of complexing agents in the citrate bath regulated the availability of Ni^2^^+^ ions at the cathode surface. This ensured a more controlled and uniform nucleation process, even at elevated temperatures, reducing the tendency to anisotropic growth. As a result, nickel deposits from citrate baths typically display finer grains and a nanocrystalline structure, leading to an increased specific surface area.

The EDAX analysis confirmed the presence of nickel, carbon, and oxygen for all analyzed samples. The carbon originated from the graphite substrate, while the oxygen content was higher in nickel deposits from the acidic Watts bath compared to those from the alkaline citrate bath. This suggests a pronounced incorporation of oxidized species, such as nickel hydroxide or nickel oxide, in deposits obtained from the Watts bath. In contrast, the alkaline citrate bath contained citrate ligands, acting as complexing agents, stabilizing nickel ions, and reducing their direct hydrolysis or oxide formation, leading to lower oxygen incorporation in the deposit.

### 3.3. Electrocatalytic Activity of Ni-PGE Towards Hydrogen Evolution Reaction

**Figure 4:**
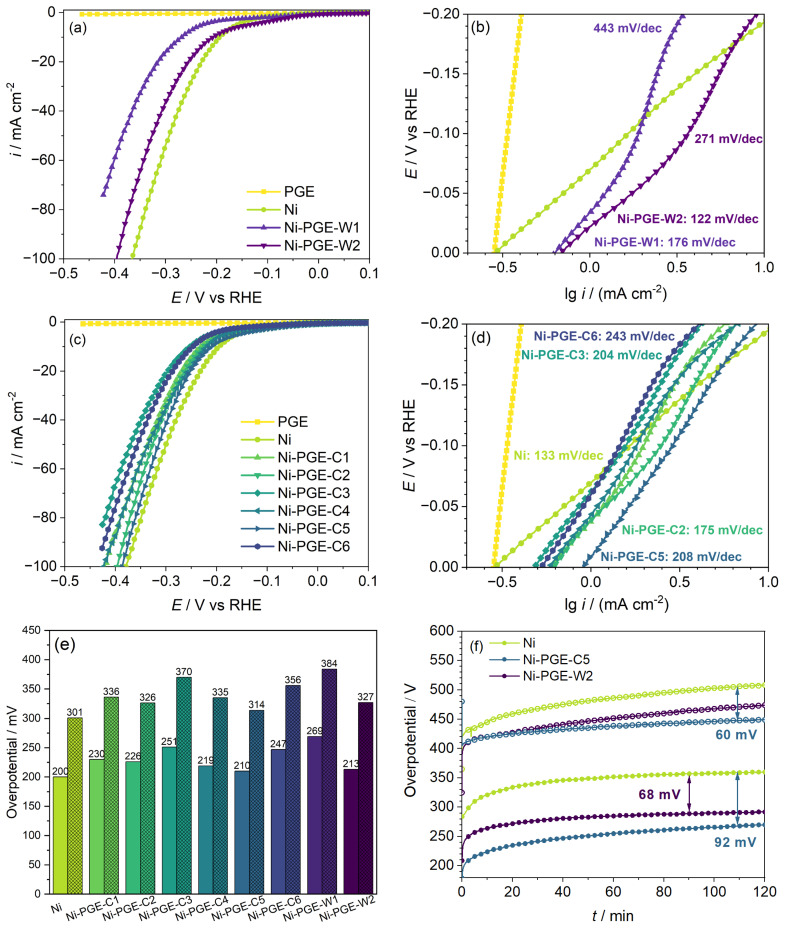
Performance of Ni-PGE for HER in 1 M KOH: polarization curves at 10 mV s^−1^ scan rate and Tafel plots for Ni-PGE obtained by pulsed current electrodeposition from (**a**,**b**) Watts bath and (**c**,**d**) citrate bath; (**e**) overpotential required to attain a current density of 10 and 50 mA cm^−2^ for Ni and Ni-PGE; (**f**) chronopotentiometric stability test of Ni, Ni-PGE-W2, and Ni-PGE-C5 at a current density of 10 mA cm^−2^ (closed symbols) and 100 mA cm^−2^ (open symbols). Polarization curves were *iR*-corrected using the ohmic resistances obtained from impedance data.

Figure 4 shows the performance of Ni-PGE for HER in alkaline solution, which was evaluated based on linear potentiodynamic polarization curves, Tafel plots, the overpotential value required to reach a current density of 10 and 50 mA cm^−2^, and stability tests at a current density of 10 and 100 mA cm^−2^, respectively. The polarization curves obtained for Ni deposits from the Watts (Figure 4a) and citrate baths (Figure 4c) reveal that unlike pencil graphite, which showed no electrocatalytic activity for HER, all the Ni-PGE showed good electrocatalytic activity. For both types of Ni deposits obtained from the Watts and citrate baths, increasing the deposition temperature from 25 to 40 °C had a favorable effect on the performance of the electrodes at both 25 and 50 mA cm^−2^ applied pulsed current densities. Further increasing the deposition temperature to 60 °C for the citrate bath negatively affected the electrocatalytic activity. In conclusion, we established that the optimal deposition temperature is 40 °C.

Tafel plots for HER of Ni-PGEs from the Watts (Figure 4b) and citrate baths (Figure 4d) allowed for a quantitative assessment of the electrocatalytic activity for HER, using the kinetic parameters (*i_o_*—exchange current density; *b*—Tafel slope). The values of the kinetic parameters, together with the overpotential necessary to reach current densities of 10, 50, and 100 mA cm^−2^, respectively, are summarized in Table 3.

The exchange current density *i_o_* is a parameter used to evaluate the reaction rate at the equilibrium potential. According to the data presented in Table 3, all Ni-PGEs have higher exchange current density values than Ni. However, these values are apparent, as they depend on the specific surface area of the electrodes. Another essential parameter for assessing the electrochemical performance of electrocatalysts away from equilibrium is the overpotential required to achieve a specific current density. Typically, the overpotentials necessary to reach current densities of 10 or 100 mA cm^−2^ are reported. Figure 4e provides an overview of the overpotentials needed to attain 10 and 50 mA cm^−2^ for all Ni-PGEs. The Ni wire electrode, used as reference, exhibited an overpotential of approximately 200 mV at 10 mA cm^−2^ and 300 mV at 50 mA cm^−2^. In comparison, Ni-PGE electrodes generally showed slightly higher overpotentials, depending on the current density, bath composition, and temperature applied during electrodeposition of nickel. Within the citrate bath series, a higher electrodeposition current density resulted in modified electrodes with lower HER overpotential, indicating improved catalytic activity. Additionally, increasing the deposition temperature from 25 °C to 40 °C reduced the HER overpotential, enhancing performance. However, a further increase in temperature during electrodeposition to 60 °C resulted in modified electrodes with higher HER overpotentials compared to those obtained at 25 and 40 °C. This suggests the existence of an optimal temperature range (e.g., around 40 °C) where nickel electrodeposition yields the most favorable microstructure and electrochemical performance. Beyond this range, further increase in temperature negatively affected HER efficiency. Among these electrodes, Ni-PGE-C5 exhibited the lowest overpotential (210 mV at 10 mA cm^−2^ and 314 mV at 50 mA cm^−2^), making it the most effective catalyst in this series. Conversely, Ni-PGE-C3 displayed the highest overpotential (251 mV at 10 mA cm^−2^ and 370 mV at 50 mA cm^−2^), indicating reduced HER efficiency. For electrodes obtained from the Watts bath, Ni-PGE-W1 demonstrated the highest overpotential overall, indicating that room temperature deposition from the Watts bath negatively affects HER performance. However, Ni-PGE-W2 performed relatively better, with an overpotential of 213 mV at 10 mA cm^−2^ and 327 mV at 50 mA cm^−2^, making it comparable to Ni-PGE-C5. Figure 4f presents the stability tests conducted at a constant current density of 10 and 100 mA cm^−2^ for the best-performing electrodes. The results show a marked increase in overpotential at 10 mA cm^−2^ during the first 20 min, followed by a slower, more gradual increase before stabilizing after approximately 2 h. Among the tested materials, Ni exhibited the highest overpotential for HER at around 360 mV. In contrast, Ni-PGE-W2 achieved a reduction of approximately 68 mV, while Ni-PGE-C5 exhibited an even greater decrease of about 92 mV, indicating superior catalytic performance. The trend in overpotentials was maintained even at a higher current density of 100 mA cm^−2^, although the difference between Ni and Ni-PGE-C5 is smaller. The results of the stability tests indicate a different order of electrocatalytic activity than that derived from the polarization curves. It is interesting to note that, in the polarization curves, the initial overpotential for Ni is lower (200 mV) compared to Ni-PGE-C5 (210 mV) and Ni-PGE-W2 (213 mV). This suggests that under short-term conditions, Ni appears slightly better at initiating HER. On the contrary, the stability test showed that over time, Ni exhibited the highest overpotential (360 mV), while Ni-PGE-C5 and Ni-PGE-W2 maintained significantly lower values (270 mV and 290 mV, respectively). The discrepancies can be explained by the differences between the two methods. The polarization curves reflect instantaneous performance, and they do not capture long-term stability or electrode degradation. The stability test highlights how the electrodes perform under prolonged electrochemical operation. The increased overpotential of Ni suggests surface passivation or catalyst site deactivation during long-term tests. Ni-PGE-C5 and Ni-PGE-W2 exhibited lower overpotentials, implying better electrocatalytic activity for HER. Although Ni initially showed a lower overpotential in the polarization curves, it experienced significantly higher overpotentials over time, making it less effective for prolonged HER applications. In contrast, Ni-PGE-C5, despite having a slightly higher initial overpotential, outperformed Ni in stability, making it the superior electrode for long-term HER performance.

According to the data presented in Table 3, the Tafel slope obtained for Ni is consistent with values reported in previous studies [39,40] and closely approaches the theoretical value of −118 mV dec^−1^. This suggests that the charge transfer step (Volmer reaction) is likely the rate-determining step on Ni. In contrast, the Ni-PGE electrodes exhibited higher Tafel slopes, ranging from −175 to −243 mV dec^−1^. Tafel slopes around −200 mV dec^−1^ are commonly reported for Ni-based electrocatalysts under similar conditions [41,42]. Notably, a change in Tafel slope was observed at overpotentials exceeding −100 mV, particularly for Ni-PGE samples deposited from the Watts bath, indicating possible changes in the reaction mechanism. Additionally, both the exchange current density (*i*_o_) and Tafel slope (*b*) were influenced by the electrodeposition temperature, with the highest *i*_o_ and lowest *b* values consistently observed at 40 °C across all series.

**Figure 5:**
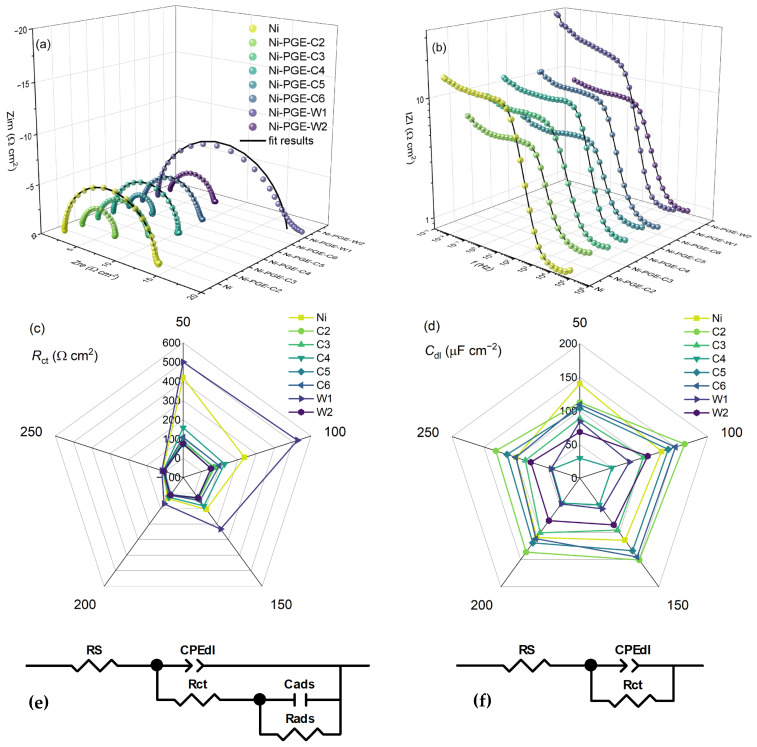
Electrochemical impedance data of Ni and Ni-PGE in 1 M KOH measured at an overpotential of −250 mV. (**a**) Nyquist and (**b**) Bode plots; (**c**) charge transfer resistance and (**d**) double-layer capacitance as a function of overpotential; (**e**) equivalent electrical circuit used to model the impedance data at low and (**f**) high overpotentials.

The performance of Ni-PGEs was further investigated using electrochemical impedance spectroscopy. To gain deeper insight into their behavior and underlying HER mechanism, EIS measurements were carried out at different overpotential values. The results are presented in Figure 5 as complex plane Nyquist plots (Figure 5a) and impedance magnitude versus frequency plots (Figure 5b), both recorded at the highest overpotential of −250 mV. Additionally, the dependence of charge transfer resistance (Figure 5c) and double-layer capacitance (Figure 5d) versus overpotential is shown. The Nyquist plots for nickel exhibit a single semicircle across all measured overpotentials (Figure S1). In contrast, the Nyquist plots for Ni-PGE display an overpotential dependent shape, exemplified for Ni-PGE-C5 in Figure S3. At low overpotentials (bellow −100 mV), the complex plane plots feature a depressed semicircle at high frequencies and either an overlapping semicircle or a tail at low frequencies. At higher overpotentials (above −100 mV), only a single semicircle is observed. The presence of a single time constant in the phase angle Bode plots for Ni can be observed in Figure S2, while the appearance of two time constants at low overpotentials for Ni-PGE-C5 is evident in Figure S4. This behavior is consistent with the differences observed in the Tafel slopes; i.e., Ni exhibits a single Tafel slope, while for Ni-PGEs, the Tafel slope depends on the overpotential. To model this behavior, two different equivalent electric circuits were employed, as given in Figure 5e,f. Both EECs comprise the solution resistance (*R*_S_) in series with a parallel connection of the double-layer capacitance (*C*_dl_, represented as a constant phase element CPE) and the charge transfer resistance (*R*_ct_). For low overpotentials, a second parallel branch composed of *R*_ads_ and *C*_ads_ was added to model the low-frequency behavior associated with adsorption phenomena. Here, *R_ads_* represents the mass transfer resistance of the adsorbed hydrogen species, while *C_ads_* accounts for the pseudo-capacitance of the adsorbed hydrogen layer [43]. The high-frequency semicircle in the Nyquist plot corresponds to double-layer charging and fast charge transfer (*C*_dl_-*R*_ct_), whereas the low-frequency semicircle or tail is attributed to mass transport of adsorbed hydrogen (*C*_ads_-*R*_ads_). The obtained impedance spectra for Ni-PGE at low overpotentials align well with this mechanism, revealing a depressed semicircle at high frequencies followed by an overlapping semicircle or a tail at low frequencies. The diameter of both semicircles is overpotential-dependent, confirming they are both related to HER kinetics.

Several studies have demonstrated that at lower cathodic overpotentials, two relaxation frequencies in impedance spectra allow separation between different steps of the HER mechanism, highlighting the contribution of hydrogen adsorption processes in this potential range [41,42,44,45]. In the Nyquist plots, the low-frequency semicircle is ascribed to the charge transfer process, e.g., Volmer reaction in the HER mechanism, while the high-frequency semicircle could be associated with the mass transfer processes of the adsorbed species at the cathode [43,46].

The Nyquist plots in Figure 5a reveal the presence of a single depressed semicircle, whose diameter corresponds to the charge transfer resistance. It can be observed that all Ni-PGE samples deposited at 40 °C, i.e., Ni-PGE-C2, Ni-PGE-C5, and Ni-PGE-W2, consistently show lower-diameter semicircles compared to samples deposited at different temperatures and Ni. These samples also present the lowest absolute impedance values in the Bode plots illustrated in Figure 5b.

The impedance parameters obtained by fitting the experimental data obtained at the highest overpotential of −250 mV, using the EEC given in Figure 5f, are summarized in Table 4. The impedance parameters obtained at lower overpotentials are given in the Supplementary Material in Table S1 (*η* = −200 mV), Table S2 (*η* = −150 mV), Table S3 (*η* = −100 mV), and Table S4 (*η* = −50 mV), respectively, along with their associated errors and the goodness-of-fit indicators, represented by chi-square values. The low errors and small chi-square values indicate a high-quality fit between the experimental data and the proposed equivalent circuit models.

The double-layer capacitance was calculated from the constant phase element parameters, whose impedance is given by

*Z*_CPE_ = *T*^−1^(j*ω*)^−*n*^ (1)

by applying Brug’s relationship [47]:

*C*_dl_ = *T*^1/*n*^ (*R*_S_^−1^ + *R*_ct_^−1^)^(*n*−1)/*n*^ (2)

where *T* is a frequency independent parameter (F cm^−2^ s^n−1^) related to the capacitance, *j* is the imaginary unit, *ω* is the angular frequency (rad s^−1^), and *n* is a dimensionless parameter between 0 and 1 that describes the constant phase angle of CPE and is equal to −(n*90) [48].

Based on the impedance data presented in Table 4, it can be observed that Ni-PGE-C5 shows the lowest *R_ct_* value, indicating the most effective charge transfer kinetics among the tested electrodes. Notably, increasing the deposition temperature for both the citrate and Watts baths led to an increase in the surface roughness, as evidenced by the decrease in parameter *n*, which is typically associated with surface inhomogeneities. However, the trends in double-layer capacitance (*C*_dl_) and charge transfer resistance (*R*_ct_) did not follow this behavior. Instead, the data suggest that a deposition temperature of 40 °C yields optimal electrocatalytic performance, characterized by the highest *C*_dl_ and the lowest *R*_ct_.

The results presented in Figure 5c and Tables S1–S4 show that *R*_ct_ values decrease with increasing overpotential. Across all overpotentials, the samples obtained by electrodeposition at 50 mA cm^−2^ and 40 °C (Ni-PGE-C5 and Ni-PGE-W2) consistently exhibited the lowest *R*_ct_ values, indicating faster charge transfer kinetics. Furthermore, comparing the samples obtained by electrodeposition at 50 mA cm^−2^ and 25 °C from the citrate and Watts baths (Ni-PGE-C4 and Ni-PGE-W1) reveals that the electrocatalyst obtained from the citrate bath always presented lower *R*_ct_ values than its Watts bath counterpart, highlighting the favorable effect of the citrate bath on electrocatalyst performance. Analysis of the double-layer capacitance values presented in Figure 5d and Tables S1–S4 indicates that *C*_dl_ decreased with increasing overpotential. This can be explained by the reduction in the electrochemical active surface area caused by gas bubble accumulation and blocking effects resulting from intensified hydrogen evolution at more negative overpotentials [49,50]. The highest *C*_dl_ values were observed for Ni-PGE-C2 (electrodeposited at 25 mA cm^−2^ and 40 °C); however, these values do not correspond to the lowest *R*_ct_ values. In contrast, Ni-PGE-C4 (electrodeposited at 50 mA cm^−2^ and 25 °C) showed the lowest *C*_dl_ values and were equivalent to the lowest active surface area.

For a clearer overview on Ni-PGE electrocatalysts performance for HER, Table 5 gives a comparison of kinetic parameters related to HER for the Ni-PGE electrodes developed in this work, alongside different literature-reported Ni-based electrocatalysts obtained by potentiostatic or galvanostatic electrodeposition from different types of baths.

The Ni-PGE-C5 electrode, obtained via pulsed current electrodeposition from a citrate bath at 50 mA cm^−2^ and 40 °C, demonstrated excellent electrocatalytic activity towards HER. It achieved a high exchange current density comparable to that of more complex electrocatalysts, such as NiMo alloy obtained by galvanostatic electrodeposition at 30 °C from a citrate bath with pH around 10 [50,52] or by pulsed current electrodeposition at 40 °C from a citrate bath with pH = 9 [51]. Despite having slightly lower *i_o_* values and a higher Tafel slope than Ni thin film deposited at a constant potential from a citrate bath at pH = 1.47 under the influence of an external magnetic field [28], it compensated by exhibiting lower overpotentials at current densities of 10 and 100 mA cm^−2^. Furthermore, in terms of exchange current density, Ni-PGE-C5 significantly outperformed Ni deposited from the Watts bath at a constant current density of 50 mA cm^−2^ and room temperature [55] as well as nickel composite nanoparticles consisting of Ni, NiO, and Ni(OH)_2_ prepared by cyclic voltammetry [53]. These results highlight the potential of Ni-PGE-C5 as a cost-effective and efficient HER electrocatalyst for alkaline water electrolysis, particularly considering its simple preparation method.

## 4. Conclusions

Nickel electrodeposition from both the acidic Watts and alkaline citrate baths was performed by pulsed current method on pencil graphite substrate at different current densities and temperatures to investigate the effect on the electrocatalytic performance for HER in 1 M KOH solution. The obtained results showed the following:

(1) The morphology of the Ni deposit strongly depends on the type of bath used for electrodeposition, revealing a heterogeneous, layered structure with dendritic and filament-like growths for the Watts bath and a homogeneous globular morphology, with structures approximately 1 µm in size for the citrate bath;
(2) Linear polarization curves for HER in 1 M KOH solution indicate that increasing the electrodeposition temperature from 25 to 40 °C improves the performance of Ni-PGE obtained from both the Watts and citrate baths at both applied pulsed current densities. Further increasing the electrodeposition temperature to 60 °C has a negative effect, resulting in Ni electrocatalysts with lower HER performance;
(3) Kinetic parameters extracted from the Tafel plots demonstrate higher exchange current density values for all Ni-PGE electrocatalysts as compared to metallic Ni, which is attributed to an increased active surface area;
(4) Chronopotentiometric measurements of HER overpotentials indicate that Ni-PGE-C5 electrocatalyst obtained from the citrate bath at 40 °C and 50 mA cm^−2^ achieves the best HER performance over time, showing an important overpotential reduction compared to metallic Ni;
(5) EIS analysis revealed the lowest charge transfer resistance for Ni-PGE-C5, indicating enhanced HER kinetics and better electrocatalytic properties.

These results highlight the optimal electrodeposition parameters regarding the bath type, temperature, and current density required to design efficient HER electrocatalysts for applications in alkaline water electrolysis.

## Figures and Tables

**Table 1:** The composition of the citrate and Watts baths used for the deposition of nickel.

Bath Type	Composition	Concentration	pH
Citrate	Na_3_C_6_H_5_O_7_·5H_2_O NiSO_4_·6 H_2_O	0.3 mol L^−1^ 0.2 mol L^−1^	8
Watts	NiSO_4_·6 H_2_O NiCl_2_·6 H_2_O H_3_BO_3_	300 g L^−1^ 45 g L^−1^ 30 g L^−1^	2

**Table 2:** Electrodeposition parameters used for preparation of Ni-PGE.

Electrode	Pulsed Current Density (mA cm^−2^)	Temperature (°C)	Electrodeposition Conditions
Ni-PGE-W1	50	25	Watts bath pH = 2
Ni-PGE-W2	50	40	
Ni-PGE-C1	25	25	Citrate bath pH = 8
Ni-PGE-C2	25	40	
Ni-PGE-C3	25	60	
Ni-PGE-C4	50	25	
Ni-PGE-C5	50	40	
Ni-PGE-C6	50	60	

**Table 3:** Values of kinetic parameters for HER on Ni and Ni-PGE in 1 M KOH solution.

Electrode	*i*_o_ (mA cm^−2^)	*b* (mV dec^−1^)	*η* @ 10 mA cm^−2^	*η* @ 50 mA cm^−2^	*η* @ 100 mA cm^−2^
Ni	0.28	−133	−200	−301	−380
Ni-PGE-W1	0.63	−176	−269	−384	–
Ni-PGE-W2	0.67	−122	−213	−327	−396
Ni-PGE-C1	0.60	−218	−230	−336	−420
Ni-PGE-C2	0.61	−175	−226	−326	−396
Ni-PGE-C3	0.48	−204	−251	−370	–
Ni-PGE-C4	0.60	−218	−219	−335	−422
Ni-PGE-C5	0.93	−208	−210	−314	−386
Ni-PGE-C6	0.53	−243	−247	−356	–

**Table 4:** Impedance parameters for HER on Ni and Ni-PGE in 1 M KOH solution at the highest overpotential of −250 mV, obtained by fitting the impedance data to the model in Figure 5f.

Electrode	*R*_S_ (Ω cm^2^)	CPE-T (F cm^−2^ s^n−1^)	*n*	*R*_ct_ (Ω cm^2^)	*C*_dl_ (µF cm^−2^)	*Chi^2^*
Ni	1.3 (0.7%)	1.49 × 10^−4^ (6.2%)	0.95 (0.8%)	5.0 (0.8%)	99.8	3.36 × 10^−3^
Ni-PGE-C2	1.1 (0.7%)	2.99 × 10^−4^ (6.1%)	0.90 (0.8%)	4.7 (0.9%)	131.4	3.39 × 10^−3^
Ni-PGE-C3	1.0 (0.7%)	3.77 × 10^−4^ (4.6%)	0.84 (0.7%)	6.9 (0.8%)	85.4	1.97 × 10^−3^
Ni-PGE-C4	1.0 (0.8%)	7.36 × 10^−5^ (4.9%)	0.95 (0.6%)	8.8 (0.8%)	43.5	2.00 × 10^−3^
Ni-PGE-C5	1.1 (0.6%)	2.47 × 10^−4^ (6.2%)	0.91 (0.8%)	3.5 (0.9%)	113.9	1.96 × 10^−3^
Ni-PGE-C6	0.9 (0.9%)	3.82 × 10^−4^ (5.2%)	0.85 (0.7%)	8.0 (1.0%)	100.1	2.95 × 10^−3^
Ni-PGE-W1	1.1 (0.8%)	8.63 × 10^−5^ (4.0%)	0.93 (0.5%)	16.7 (0.7%)	44.8	2.50 × 10^−3^
Ni-PGE-W2	0.9 (0.8%)	3.74 × 10^−4^ (4.9%)	0.85 (0.7%)	6.3 (0.8%)	76.7	2.12 × 10^−3^

**Table 5:** Comparative data on the kinetic parameters for HER and overpotential at 10 and 100 mA cm^−2^ for different Ni-based electrocatalysts.

Electrode	**Deposition Bath/pH**	Testing Solution	*i_o_* (mA cm^−2^)	*b* (mV)	*η* @ 10 mA cm ^−2^	*η* @ 100 mA cm ^−2^	Reference
Ni-PGE-C5	Citrate/pH = 8	1 M KOH	0.93	−208	−210	−392	This work
Ni-PGE-W2	Watts/pH = 2	1 M KOH	0.67	−122	−213	−432	This work
Ni thin film	Citrate/pH = 1.47	1 M NaOH	1.98	−118	−231	over −400 *	[28]
NiMo-1	Citrate/pH = 10.5	1 M NaOH	1.0	−192	−185 *	−384	[50]
NiMo30%	Citrate/pH = 9	1 M KOH	0.487	−124	−180	n/a	[51]
NiMo	Citrate/pH = 10	1 M KOH	0.93	−152	−154	n/a	[52]
Ni/NiO/Ni(OH)_2_	NiCl_2_/acid	0.1 M KOH	0.16	−118	−210	n/a	[53]
Ni/NiO/Ni(OH)_2_	NiCl_2_/acid	1 M KOH	0.057	−88	−197	n/a	[53]
Ni/NiO_x_	Ni oxidation	1 M NaOH	0.0365	n/a	n/a	n/a	[54]
Ni	Watts/pH = 3.5	1 M NaOH	0.012	−93	−263	−350	[55]

* estimated from polarization curves.

## Data Availability

The original contributions presented in this study are included in the article and Supplementary Materials. Further inquiries can be directed to the corresponding author.

## Supplementary Materials

The following supporting information can be downloaded at https://www.mdpi.com/article/10.3390/ma18122775/s1. Figure S1: Nyquist plots of Ni in 1 M KOH measured at different overpotentials; Figure S2: Bode plots of Ni in 1 M KOH measured at different overpotentials; Figure S3: Nyquist plots of Ni-PGE-C5 in 1 M KOH measured at different overpotentials; Figure S4: Bode plots of Ni-PGE-C5 in 1 M KOH measured at different overpotentials; Table S1: Impedance parameters for HER on Ni and Ni-PGE in 1 M KOH solution at an overpotential of −200 mV obtained by fitting the impedance data to the model in Figure 5f; Table S2: Impedance parameters for HER on Ni and Ni-PGE in 1 M KOH solution at an overpotential of −150 mV obtained by fitting the impedance data to the model in Figure 5f; Table S3: Impedance parameters for HER on Ni and Ni-PGE in 1 M KOH solution at an overpotential of −100 mV obtained by fitting the impedance data to the model in Figure 5e; Table S4: Impedance parameters for HER on Ni and Ni-PGE in 1 M KOH solution at an overpotential of −50 mV obtained by fitting the impedance data to the model in Figure 5e.

## Abbreviations

The following abbreviations are used in this manuscript:

*C*_dl_	Double-layer capacitance
*i*_o_	Exchange current density
*R*_ct_	Charge transfer resistance
*R*_s_	Solution resistance
AC	Alternating current
CP	Chronopotentiometry
CPE	Constant phase element
EDAX	Energy dispersive X-ray analysis
EEC	Equivalent electric circuits
EIS	Electrochemical impedance spectroscopy
FE-SEM	Field emission scanning electron microscopy
HER	Hydrogen evolution reaction
LSV	Linear sweep voltammetry
OER	Oxygen evolution reaction
PC	Pulsed current
PGE	Pencil graphite electrode
RHE	Reversible hydrogen electrode

## References

[1] McCrory C.C.L., Jung S., Ferrer I.M., Chatman S.M., Peters J.C., Jaramillo T.F. (2015). Benchmarking Hydrogen Evolving Reaction and Oxygen Evolving Reaction Electrocatalysts for Solar Water Splitting Devices. J. Am. Chem. Soc..

[2] Ramakrishnan S., Vijayapradeep S., Selvaraj S.C., Huang J., Karthikeyan S., Gutru R., Logeshwaran N., Miyazaki T., Mamlouk M., Yoo D.J. (2024). An efficient cathode electrocatalyst for anion exchange membrane water electrolyzer. Carbon.

[3] Gebremariam G.K., Jovanović A.Z., Pašti I.A. (2023). Kinetics of Hydrogen Evolution Reaction on Monometallic Bulk Electrodes in Various Electrolytic Solutions. Catalysts.

[4] Zhu Y., Liu T., Li L., Song S., Ding R. (2018). Nickel-based electrodes as catalysts for hydrogen evolution reaction in alkaline media. Ionics.

[5] Harada Y., Hua Q., Harris L.C., Yoshida T., Gewirth A.A. (2024). Electrodeposition of Fractal Structured Nickel for Hydrogen Evolution Reaction in Alkaline. ChemElectroChem.

[6] Jawhari A.H., Hasan N. (2023). Nanocomposite Electrocatalysts for Hydrogen Evolution Reactions (HERs) for Sustainable and Efficient Hydrogen Energy—Future Prospects. Materials.

[7] Cieluch M., Kazamer N., Böhm L., Sanden S., Zerebecki S., Wirkert F., Apfel U., Brodmann M. (2025). Effect of Electrolyte pH in Additive-Free NiFe Catalyst Electrodeposition for Electro-Catalytic OER Applications. ChemElectroChem.

[8] Raja M.A., Arumainathana S. (2019). Comparative study of hydrogen evolution behavior of Nickel Cobalt and Nickel Cobalt Magnesium alloy film prepared by pulsed electrodeposition. Vacuum.

[9] Feng Y., Guan Y., Zhou E., Zhang X., Wang Y. (2022). Nanoscale Double-Heterojunctional Electrocatalyst for Hydrogen Evolution. Adv. Sci..

[10] Li J., Chu D., Poland C., Smith C., Nagelli E.A., Jaffett V. (2025). XPS Depth Profiling of Surface Restructuring Responsible for Hydrogen Evolution Reaction Activity of Nickel Sulfides in Alkaline Electrolyte. Materials.

[11] Bhat K.S., Nagaraja H. (2018). Nickel selenide nanostructures as an electrocatalyst for hydrogen evolution reaction. Int. J. Hydrogen Energy.

[12] Liu G., Hou F., Peng S., Wang X., Fang B. (2022). Synthesis, Physical Properties and Electrocatalytic Performance of Nickel Phosphides for Hydrogen Evolution Reaction of Water Electrolysis. Nanomaterials.

[13] Danish M.S.S. (2023). Exploring metal oxides for the hydrogen evolution reaction (HER) in the field of nanotechnology. RSC Sustain..

[14] Zhu Y., Lin Q., Zhong Y., Tahini H.A., Shao Z., Wang H. (2020). Metal Oxide-Based Materials as an Emerging Family of Hydrogen Evolution Electrocatalysts. Energy Environ. Sci..

[15] Huo L., Jin C., Jiang K., Bao Q., Hu Z., Chu J. (2022). Applications of Nickel-Based Electrocatalysts for Hydrogen Evolution Reaction. Adv. Energy Sustain. Res..

[16] Sadhanala H.K., Perelshtein I., Gedanken P.A. (2020). Boosting Electrocatalytic Hydrogen Evolution of Nickel foam Supported Nickel Hydroxide by Ruthenium Doping. Chem. Sel..

[17] Wang H., Xiong J., Cheng X., Fritz M., Ispas A., Bund A., Chen G., Wang D., Schaaf P. (2020). Ni3N-Coated Ni Nanorod Arrays for Hydrogen and Oxygen Evolution in Electrochemical Water Splitting. ACS Appl. Nano Mater..

[18] Faid A.Y., Barnett A.O., Seland F., Sunde S. (2018). Highly Active Nickel-Based Catalyst for Hydrogen Evolution in Anion Exchange Membrane Electrolysis. Catalysts.

[19] Shen Y., Wu P., Wang C., Yuan W., Yang W., Shang X. (2023). Electrodeposition of amorphous Ni-Fe-Mo composite as a binder-free and high-performance electrocatalyst for hydrogen generation from alkaline water electrolysis. Int. J. Hydrogen Energy.

[20] Fernández-Valverde S., Ordóñez-Regil E., Cabañas-Moreno J.G., Solorza-Feria O. (2019). Electrochemical Behavior of Ni-Mo Electrocatalyst for Water Electrolysis. J. Mex. Chem. Soc..

[21] Deng Y., Lai W., Xu B. (2020). A Mini Review on Doped Nickel-Based Electrocatalysts for Hydrogen Evolution Reaction. Energies.

[22] Wang X., Tian H., Zhu L., Li S., Cui X. (2024). Synergetic Catalytic Effect between Ni and Co in Bimetallic Phosphide Boosting Hydrogen Evolution Reaction. Nanomaterials.

[23] Li R., Zang P.J., Li P.W., Li J., Zou Q., Zhou S., Su J., Wang P.Y. (2020). Three-Dimensional Transition Metal Phosphide Heteronanorods for Efficient Overall Water Splitting. ChemSusChem.

[24] Zhang D., Shi J., Qi Y., Wang X., Wang H., Li M., Liu S., Li C. (2018). Quasi-Amorphous Metallic Nickel Nanopowder as an Efficient and Durable Electrocatalyst for Alkaline Hydrogen Evolution. Adv. Sci..

[25] Arcas R., Koshino Y., Mas-Marza E., Tsuji R., Masutani H., Miura-Fujiwara E., Haruyama Y., Nakashima S., Ito S., Fabregat-Santiago F. (2021). Pencil graphite rods decorated with nickel and nickel–iron as low-cost oxygen evolution reaction electrodes. Sustain. Energy Fuels.

[26] Poimenidis I.A., Moustaizis S.D., Papakosta N., Tsanakas M.D., Klini A., Loukakos P.A. (2022). Electrodeposition of Ni particles on laser nanostructured electrodes for enhanced hydrogen evolution reaction. Mater. Today Proc..

[27] Xu C., Chen P., Hu B., Xiang Q., Cen Y., Hu B., Liu L., Liu Y., Yu D., Chen C. (2020). Porous nickel electrodes with controlled texture for the hydrogen evolution reaction and sodium borohydride electrooxidation. CrystEngComm.

[28] Elsharkawy S., Kutyła D., Zabinski P. (2023). The Influence of the Magnetic Field on Ni Thin Film Preparation by Electrodeposition Method and Its Electrocatalytic Activity towards Hydrogen Evolution Reaction. Coatings.

[29] Boubatra M., Azizi A., Schmerber G., Dinia A. (2012). The influence of pH electrolyte on the electrochemical deposition and properties of nickel thin films. Ionics.

[30] Wang X., Jiang M., Yang P., Zhou H., Xi W., Duan J., Ratova M., Wu D., Jiang X. (2024). Recent advances in pulsed electrochemical techniques: Synthesis of electrode materials and electrocatalytic reactions. Surf. Interfaces.

[31] Boukhouiete A., Boumendjel S., Sobhi N.E.H., Creus J. (2022). Microstructural investigation of nickel deposits obtained by pulsed current. J. Ind. Chem. Soc..

[32] Balint L.-C., Hulka I., Kellenberger A. (2022). Pencil Graphite Electrodes Decorated with Platinum Nanoparticles as Efficient Electrocatalysts for Hydrogen Evolution Reaction. Materials.

[33] Torrinha A., Amorim C.G., Montenegro M.C., Araújo A.N. (2018). Biosensing based on pencil graphite electrodes. Talanta.

[34] Habibi B., Farhadi K., Minaie E. (2024). Electrodeposited pectin/reduced carbon dots scaffold on the pencil graphite electrode as a support of electroloaded nickel nanoparticles for electrocatalytic purpose. Int. J. Hydrogen Energy.

[35] Kayan D.B., Koçak D., İlhan M., Koca A. (2017). Electrocatalytic hydrogen production on a modified pencil graphite electrode. Int. J. Hydrogen Energy.

[36] Melo L.C., de Lima-Neto P., Correia A.N. (2011). The influence of citrate and tartrate on the electrodeposition and surface morphology of Cu–Ni layers. J. Appl. Electrochem..

[37] Li C., Li X., Wang Z., Guo H. (2017). Nickel electrodeposition from novel citrate bath. Trans. Nonferrous Met. Soc. China.

[38] Bigos A., Wolowicz M., Janusz-Skuza M., Starowicz Z., Szczerba M.J., Bogucki R., Beltowska-Lehman E. (2021). Citrate-based baths for electrodeposition of nanocrystalline nickel coatings with enhanced hardness. J. Alloys Compd..

[39] Lasia A., Rami A. (1990). Kinetics of hydrogen evolution on nickel electrodes. J. Electroanal. Chem. Interfacial Electrochem..

[40] Krstajič N., Popovič M., Grgur B., Vojnovič M., Sepa D. (2001). On the kinetics of hydrogen evolution reaction on nickel in alkaline solution: Part I. The mechanism. J. Electroanal. Chem..

[41] Franceschini E.A., Lacconi G.I., Corti H.R. (2015). Kinetics of the hydrogen evolution on nickel in alkaline solution: New insight from rotating disk electrode and impedance spectroscopy analysis. Electrochim. Acta.

[42] Kellenberger A., Vaszilcsin N., Brandl W., Duteanu N. (2007). Kinetics of hydrogen evolution reaction on skeleton nickel and nickel–titanium electrodes obtained by thermal arc spraying technique. Int. J. Hydrogen Energy.

[43] Nikolic V.M., Maslovara S.L.J., Tasic G.S., Brdaric T.P., Lausevic P.Z., Radak B.B., Kaninski M.P.M. (2015). Kinetics of hydrogen evolution reaction in alkaline electrolysis on a Ni cathode in the presence of Ni–Co–Mo based ionic activators. Appl. Catal. B.

[44] Horvat-Radošević V., Kvastek K. (2003). Impedance Spectra of the Hydrogen Evolution Reaction at Low Cathodic Overpotentials. Croat. Chem. Acta.

[45] Crețu R., Kellenberger A., Vaszilcsin N. (2013). Enhancement of hydrogen evolution reaction on platinum cathode by proton carriers. Int. J. Hydrogen Energy.

[46] Choquette Y., Brossard L., Lasia A., Menard H. (1990). Study of the Kinetics of Hydrogen Evolution Reaction on Raney Nickel Composite-Coated Electrode by AC Impedance Technique. J. Electrochem. Soc..

[47] Brug G.J., van den Eeden A.L.G., Sluyters-Rehbach M., Sluyters J.H. (1984). The Analysis of Electrode Impedances Complicated by the Presence of a Constant Phase Element. J. Electroanal. Chem. Interfacial Electrochem..

[48] Gateman S.M., Gharbi O., Gomes de Melo H., Ngo K., Turmine M., Vivier V. (2022). On the use of a constant phase element (CPE) in electrochemistry. Curr. Opin. Electrochem..

[49] Perović I., Marčeta Kaninski M., Tasić G., Maslovara S., Laušević P., Seović M., Nikolić V. (2023). Enhanced Catalytic Activity and Energy Savings with Ni-Zn-Mo Ionic Activators for Hydrogen Evolution in Alkaline Electrolysis. Materials.

[50] Manazoğlu M., Hapçı G., Orhan G. (2016). Electrochemical Deposition and Characterization of Ni-Mo Alloys as Cathode for Al-kaline Water Electrolysis. J. Mater. Eng. Perform..

[51] Llorente V.B., Diaz L.A., Lacconi G.I., Abuin G.C., Franceschini E.A. (2022). Effect of duty cycle on NiMo alloys prepared by pulsed electrodeposition for hydrogen evolution reaction. J. Alloys Compd..

[52] Bao F., Kemppainen E., Dorbandt I., Bors R., Xi F., Schlatmann R., van de Krol R., Calnan S. (2020). Understanding the Hydrogen Evolution Reaction Kinetics of Electrodeposited Nickel-Molybdenum in Acidic, Near-Neutral, and Alkaline Conditions. ChemElectroChem.

[53] Tao S., Yang F., Schuch J., Jaegermann W., Kaiser B. (2018). Electrodeposition of Nickel Nanoparticles for the Alkaline Hydrogen Evolution Reaction: Correlating Electrocatalytic Behavior and Chemical Composition. ChemSusChem.

[54] Kuznetsov A.N., Oshchepkov A.G., Cherstiouk O.V., Simonov P.A., Nazmutdinov R.R., Savinova E.R., Bonnefont A. (2020). Influence of the NaOH Concentration on the Hydrogen Electrode Reaction Kinetics of Ni and NiCu Electrodes. ChemElectroChem.

[55] Solmaz R., Kardas G. (2009). Electrochemical deposition and characterization of NiFe coatings as electrocatalytic materials for alkaline water electrolysis. Electrochim. Acta.

