# Supplementary material for: Nickel Electrocatalysts Obtained by Pulsed Current Electrodeposition from Watts and Citrate Baths for Enhanced Hydrogen Evolution Reaction in Alkaline Media
Source: Materials (Basel). 2025 Jun 12;18(12):2775. doi: 10.3390/ma18122775 (PMC12195463; doi:10.3390/ma18122775)
Supplement: Supplementary file 1 [file materials-18-02775-s001.zip › materials-3661917-supplementary.pdf]

Supplementary material

Nickel Electrocatalysts Obtained by Pulsed Current Electrodeposition from Watts and Citrate Baths for Enhanced Hydrogen Evolution Reaction in Alkaline Media

Raluca Bojîncă [1], Roxana Muntean [2], Rebeca Crişan [1] and Andrea Kellenberger [1,*]

[1] Faculty of Chemical Engineering, Biotechnologies and Environmental Protection, Politehnica University Timişoara, Piata Victoriei No. 2, 300006 - Timişoara, Romania; raluca.bojinca@student.upt.ro (R.B.); andrea.kellenberger@upt.ro (A.K.)

[2] Department of Materials and Manufacturing Engineering, Politehnica University Timișoara, Piata Victoriei No. 2, 300006 - Timişoara, Romania; roxana.muntean@upt.ro

***** Correspondence: andrea.kellenberger@upt.ro; Tel.: +40-256-404176

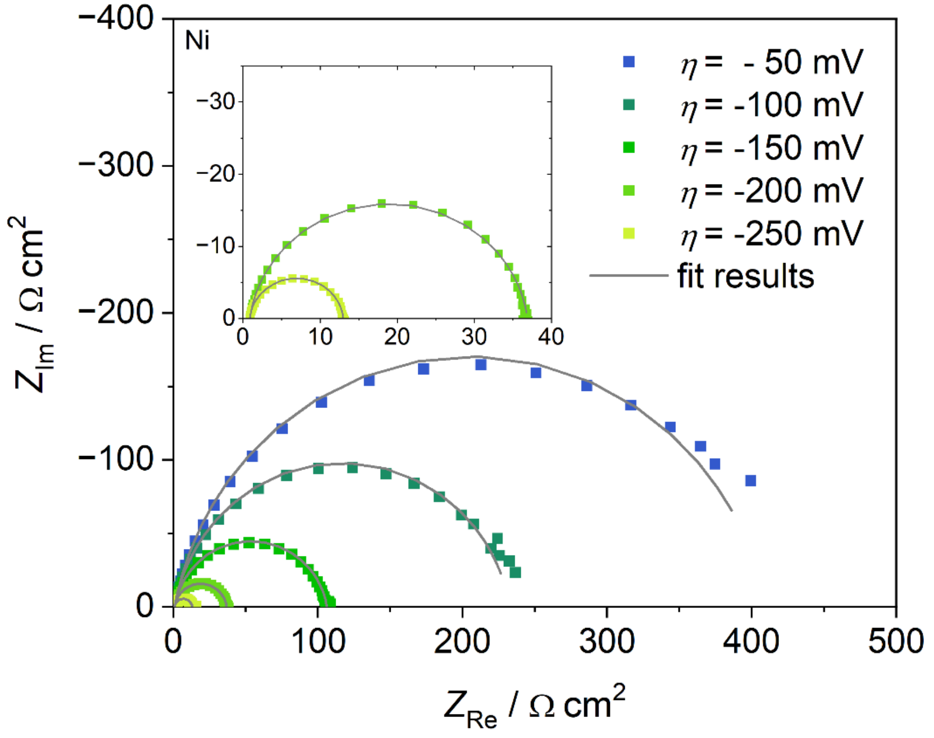

Figure S1. Nyquist plots of Ni in 1 M KOH measured at different overpotentials.

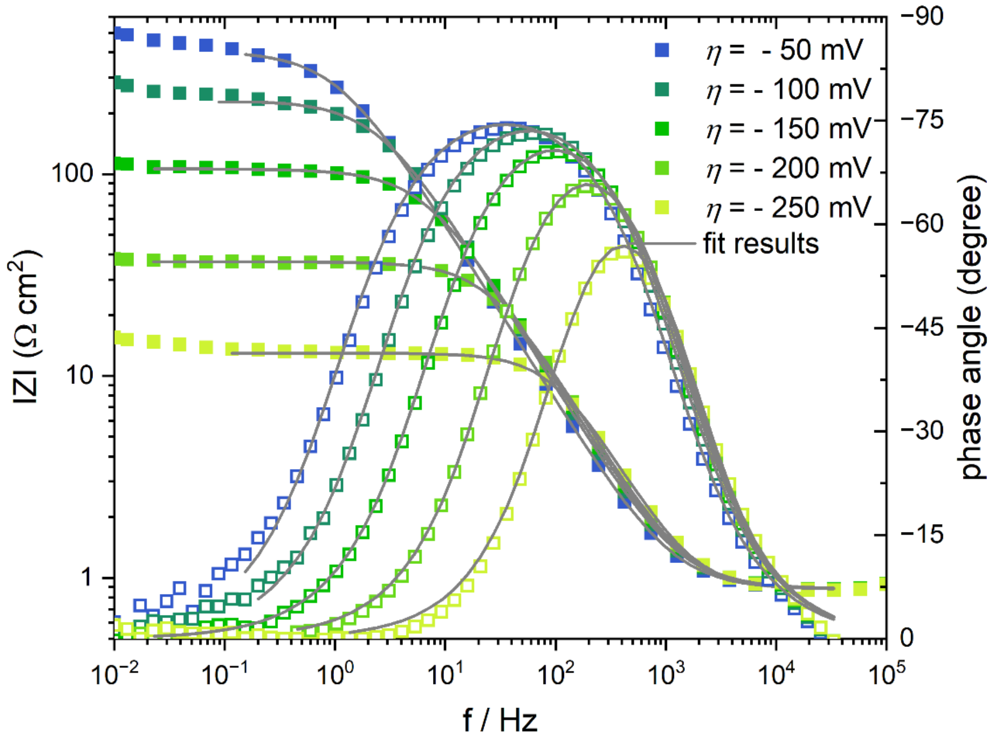

Figure S2. Bode plots of Ni in 1 M KOH measured at different overpotentials.

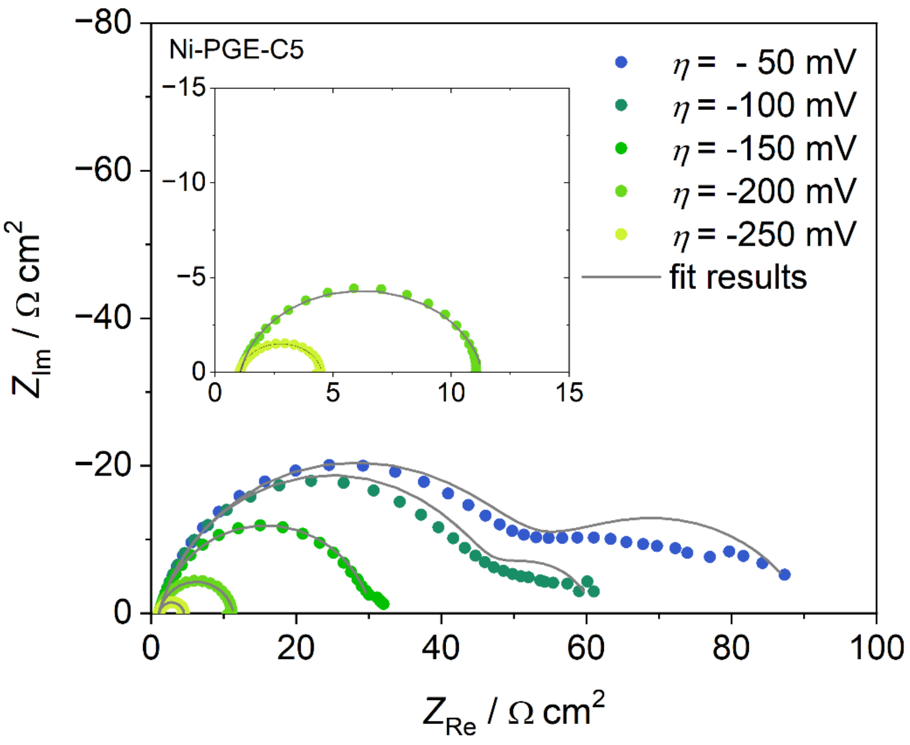

Figure S3. Nyquist plots of Ni-PGE-C5 in 1 M KOH measured at different overpotentials.

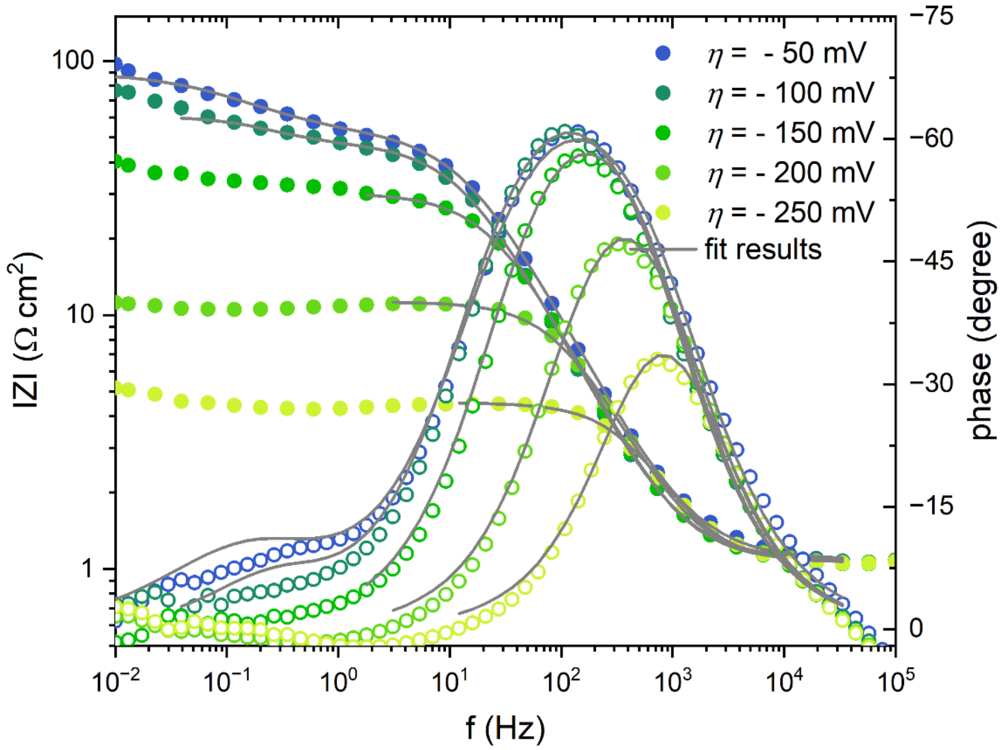

Figure S4. Bode plots of Ni-PGE-C5 in 1 M KOH measured at different overpotentials.

Table S1. Impedance parameters for HER on Ni and Ni-PGE in 1 M KOH solution at an overpotential of –200 mV obtained by fitting the impedance data to the model in Fig. 5f.

Electrode	R_S (Ω cm^2)	CPE-T (F cm^{-2} s^{n-1})	n	R_{ct} (Ω cm^2)	C_{dl} (μF cm^{-2})	Chi^2
Ni	0.9 (0.4%)	2.56×10^{-4} (1.1%)	0.92 (0.2%)	36.0 (0.3%)	124.8	5.49×10^{-4}
Ni-PGE-C2	1.1 (0.8%)	3.17×10^{-4} (4.2%)	0.90 (0.6%)	12.5 (0.9%)	136.1	3.43×10^{-3}
Ni-PGE-C3	1.1 (0.8%)	7.32×10^{-4} (3.4%)	0.78 (0.6%)	15.9 (0.8%)	100.6	2.07×10^{-3}
Ni-PGE-C4	1.0 (0.6%)	8.64×10^{-5} (2.4%)	0.94 (0.3%)	27.1 (0.5%)	45.8	1.35×10^{-3}
**Ni-PGE-C5**	**1.1 (0.7%)**	**3.18×10^{-4} (4.0%)**	**0.89 (0.6%)**	**10.3 (0.8%)**	**119.0**	**2.26×10^{-3}**
Ni-PGE-C6	0.9 (1.0%)	5.11×10^{-4} (4.3%)	0.83 (0.7%)	14.9 (1.0%)	111.4	3.73×10^{-3}
Ni-PGE-W1	1.1 (1.1%)	1.05×10^{-4} (3.3%)	0.92 (0.5%)	69.2 (0.7%)	47.3	8.16×10^{-3}
Ni-PGE-W2	0.9 (0.8%)	5.45×10^{-4} (3.4%)	0.79 (0.5%)	13.5 (0.7%)	78.2	2.30×10^{-3}

Table S2. Impedance parameters for HER on Ni and Ni-PGE in 1 M KOH solution at an overpotential of –150 mV obtained by fitting the impedance data to the model in Fig. 5f.

Electrode	R_S (Ω cm^2)	CPE-T (F cm^{-2} s^{n-1})	n	R_{ct} (Ω cm^2)	C_{dl} (μF cm^{-2})	Chi^2
Ni	0.9 (0.4%)	3.28×10^{-4} (0.8%)	0.90 (0.1%)	105.4 (0.3%)	129.7	6.96×10^{-4}
Ni-PGE-C2	1.1 (0.7%)	3.96×10^{-4} (2.6%)	0.89 (0.4%)	38.0 (0.7%)	150.6	3.57×10^{-3}
Ni-PGE-C3	1.1 (0.8%)	7.51×10^{-4} (2.6%)	0.77 (0.5%)	32.7 (0.7%)	95.4	2.47×10^{-3}
Ni-PGE-C4	1.0 (0.7%)	1.20×10^{-4} (1.9%)	0.91 (0.3%)	83.2 (0.6%)	49.2	1.91×10^{-3}
**Ni-PGE-C5**	**1.1 (0.7%)**	**4.36×10^{-4} (2.6%)**	**0.87 (0.4%)**	**29.9 (0.7%)**	**133.5**	**2.35×10^{-3}**
Ni-PGE-C6	0.9 (0.9%)	5.64×10^{-4} (2.8%)	0.85 (0.5%)	48.9 (1.1%)	145.0	3.64×10^{-3}
Ni-PGE-W1	1.1 (1.3%)	1.28×10^{-4} (3.0%)	0.92 (0.5%)	231.8 (1.0%)	56.3	9.79×10^{-3}
Ni-PGE-W2	0.9 (1.2%)	7.66×10^{-4} (3.5%)	0.77 (0.6%)	31.4 (0.8%)	85.9	6.57×10^{-3}

Table S3. Impedance parameters for HER on Ni and Ni-PGE in 1 M KOH solution at an overpotential of –100 mV obtained by fitting the impedance data to the model in Fig. 5e.

Electrode	R_S (Ω cm²)	CPE-T (F cm⁻² sⁿ⁻¹)	n	R_{ct} (Ω cm²)	C_{dl} (μF cm⁻²)	R_{ads} (Ω cm²)	C_{ads} (μF cm⁻²)	Chi^2
Ni	0.9 (0.6%)	3.69×10^{-4} (1.0%)	0.89 (0.2%)	236.7 (0.5%)	138.3	-	-	1.24×10^{-3}
Ni-PGE-C2	1.1 (0.8%)	4.86×10^{-4} (2.9%)	0.87 (0.5%)	66.2 (1.3%)	164.0	7.0 (19.2%)	7.72×10^{-2} (35.4%)	5.10×10^{-3}
Ni-PGE-C3	1.1 (0.9%)	7.63×10^{-4} (2.6%)	0.78 (0.5%)	62.6 (1.1%)	99.4	4.1 (26.5%)	3.52×10^{-1} (48.2%)	4.20×10^{-3}
Ni-PGE-C4	1.0 (0.8%)	1.58×10^{-4} (1.9%)	0.88 (0.4%)	111.4 (1.5%)	50.2	15.6 (11.8%)	5.15×10^{-3} (26.3%)	2.42×10^{-3}
**Ni-PGE-C5**	**1.1 (0.7%)**	**4.93×10^{-4} (2.9%)**	**0.86 (0.5%)**	**46.5 (1.3%)**	**138.4**	**5.7 (17.6%)**	**4.93×10^{-2} (28.5%)**	**3.27×10^{-3}**
Ni-PGE-C6	0.9 (1.0%)	6.57×10^{-4} (2.9%)	0.83 (0.5%)	79.7 (1.6%)	149.7	18.1 (10.8%)	3.22×10^{-2} (19.6%)	4.79×10^{-3}
Ni-PGE-W1	1.1 (0.7%)	1.66×10^{-4} (1.9%)	0.92 (0.3%)	379.4 (1.9%)	77.6	146.0 (5.7%)	1.64×10^{-3} (11.9%)	2.59×10^{-3}
Ni-PGE-W2	0.9 (0.8%)	8.64×10^{-4} (3.0%)	0.77 (0.5%)	43.0 (1.5%)	106.4	9.3 (8.6%)	2.67×10^{-2} (17.3%)	2.69×10^{-3}

Table S4. Impedance parameters for HER on Ni and Ni-PGE in 1 M KOH solution at an overpotential of –50 mV obtained by fitting the impedance data to the model in Fig. 5e.

Electrode	R_S (Ω cm²)	CPE-T (F cm⁻² sⁿ⁻¹)	n	R_{ct} (Ω cm²)	C_{dl} (μF cm⁻²)	R_{ads} (Ω cm²)	C_{ads} (μF cm⁻²)	Chi^2
Ni	0.9 (0.6%)	4.57×10^{-4} (0.9%)	0.88 (0.2%)	419.7 (0.7%)	160.8	-	-	1.42×10^{-3}
Ni-PGE-C2	1.0 (0.7%)	5.52×10^{-4} (2.4%)	0.82 (0.4%)	61.3 (1.1%)	112.8	15.7 (7.0%)	2.71×10^{-2} (12.4%)	2.46×10^{-3}
Ni-PGE-C3	1.1 (1.0%)	9.22×10^{-4} (2.7%)	0.75 (0.5%)	77.2 (1.3%)	89.0	13.7 (11.1%)	9.12×10^{-2} (21.1%)	3.94×10^{-3}
Ni-PGE-C4	0.9 (1.1%)	2.09×10^{-4} (2.8%)	0.81 (0.4%)	113.6 (1.3%)	29.7	45.5 (4.2%)	5.98×10^{-3} (9.4%)	3.24×10^{-3}
**Ni-PGE-C5**	**1.1 (0.7%)**	**4.89×10^{-4} (2.3%)**	**0.83 (0.4%)**	**53.9 (0.9%)**	**103.9**	**18.8 (4.5%)**	**3.15×10^{-2} (7.9%)**	**2.16×10^{-3}**
Ni-PGE-C6	0.9 (1.0%)	7.94×10^{-4} (3.0%)	0.78 (0.6%)	76.0 (1.5%)	109.4	32.9 (5.6%)	2.66×10^{-2} (10.4%)	4.38×10^{-3}
Ni-PGE-W1	1.0 (1.1%)	2.30×10^{-4} (2.3%)	0.89 (0.4%)	394.8 (2.0%)	84.2	105.1 (8.2%)	5.14×10^{-3} (18.8%)	4.54×10^{-3}
Ni-PGE-W2	0.9 (1.2%)	1.11×10^{-3} (3.5%)	0.71 (0.7%)	52.6 (1.8%)	68.7	23.3 (5.3%)	1.84×10^{-2} (10.8%)	3.72×10^{-3}
